# Association of circulating omentin level and metabolic-associated fatty liver disease: a systematic review and meta-analysis

**DOI:** 10.3389/fendo.2023.1073498

**Published:** 2023-04-17

**Authors:** Qin Zhang, Shuaihang Chen, Yani Ke, Qicong Li, Chenglu Shen, Yuting Ruan, Kaihan Wu, Jie Hu, Shan Liu

**Affiliations:** ^1^ The First Clinical Medical College of Zhejiang Chinese Medical University, Hangzhou, Zhejiang, China; ^2^ The Second Clinical Medical College of Zhejiang Chinese Medical University, Hangzhou, Zhejiang, China; ^3^ Department of Infectious Diseases, The First Affiliated Hospital of Zhejiang Chinese Medical University, Zhejiang Provincial Hospital of Chinese Medicine, Hangzhou, Zhejiang, China; ^4^ Department of Clinical Evaluation Center, The First Affiliated Hospital of Zhejiang Chinese Medical University, Zhejiang Provincial Hospital of Chinese Medicine, Hangzhou, Zhejiang, China

**Keywords:** metabolic-associated fatty liver disease (MAFLD), non-alcoholic fatty liver disease, omentin, systematic review, meta-analysis

## Abstract

**Background:**

Metabolic-associated fatty liver disease (MAFLD) is closely associated with omentin, a novel adipokine that plays a vital role in metabolic balance. The literature about the relationship between circulating omentin and MAFLD is conflicting. Therefore, this meta-analysis evaluated circulating omentin levels in patients with MAFLD compared with healthy controls to explore the role of omentin in MAFLD.

**Methods:**

The literature search was performed up to April 8, 2022, using PubMed, Cochrane Library, EMBASE, CNKI, Wanfang, CBM, Clinical Trials Database and Grey Literature Database. This meta-analysis pooled the statistics in Stata and presented the overall results using the standardized mean difference (*SMD*) and 95% confidence interval (*CI*).

**Results:**

Twelve studies with 1624 individuals (927 cases and 697 controls) were included, and all of them were case-control studies. In addition, ten of twelve included studies were conducted on Asian participants. Patients with MAFLD had significantly lower circulating omentin levels than healthy controls (*SMD*=-0.950 [-1.724, -0.177], *P*=0.016). Subgroup analysis and meta-regression demonstrated that fasting blood glucose (FBG) might be the source of heterogeneity and was inversely associated with omentin levels (coefficient=-0.538, *P*=0.009). No significant publication bias existed (*P*>0.05), and outcomes were robust in the sensitivity analysis.

**Conclusion:**

Lower circulating omentin levels were associated with MAFLD, and FBG might be the source of heterogeneity. Since Asian studies accounted for a significant portion of the meta-analysis, the conclusion might be more applicable to the Asian population. By investigating the relationship between omentin and MAFLD, this meta-analysis laid the foundation for the development of diagnostic biomarkers and treatment targets.

**Systematic review registration:**

https://www.crd.york.ac.uk/prospero/, identifier CRD42022316369.

## Introduction

Nonalcoholic fatty liver disease (NAFLD) is becoming more prevalent, alongside an increasing prevalence of obesity and diabetes mellitus, with a global incidence of 29.84% (1). Realizing the importance of the metabolic aspects of NAFLD, an expert group in 2020 recommended that the term NAFLD could be replaced by the term metabolic-associated fatty liver disease (MAFLD) (2). MAFLD is currently the most common cause of chronic liver disease, and it could develop into cirrhosis, liver failure, or hepatocellular carcinoma (3, 4). MAFLD is a global public health issue that causes systemic complications related to metabolic syndrome (MetS) or cardiovascular disease, besides hepatic impairment. Clustering of clinical studies has shown that MetS is the most characteristic feature of MAFLD and a critical contributor to disease progression (5). Adipose tissue dysfunction, the release of lipotoxic lipids, and oxidative stress also indicate crosstalk between physical metabolism and MAFLD (6). These metabolic insights make it feasible to improve diagnostic methods and unravel potential therapeutic targets for MAFLD. Regarding the diagnosis of MAFLD, histopathological biopsy remains the gold standard, though invasiveness, risk, and cost are related problems (7). Thus, there is a need for a noninvasive and precise diagnostic biomarker for MAFLD to monitor high-risk individuals and facilitate early diagnosis. As for the treatment of MAFLD, dietary modification and exercise are the mainstay treatments of MAFLD. New drugs targeting adipocyte dysfunction and insulin resistance have also been developed (8, 9).

Omentin, a secreted protein consisting of 313 amino acids, was first identified from an omental fat cDNA library in 2006 by Yang et al.. It was found to be potentially associated with insulin resistance (10). Omentins include omentin-1 and omentin-2, and omentin-1 is the major circulating form. In addition, omention-1 was also the main target of various research studies (11). In addition to mediating insulin resistance, previous studies have shown that omentin-1 played a crucial role in anti-inflammation, anti-oxidation, and regulation of apoptosis (12–14). It has been demonstrated that decreased circulating omentin levels are associated with MetS as well as MetS comorbidity with obesity, carotid atherosclerosis, and hypertension (15–18). Another meta-analysis found that the omentin levels in patients with type 2 diabetes mellitus (T2DM) were significantly lower than those in controls, and lower omentin levels increased the risk of complications in patients with diabetes (19, 20). Therefore, omentin-1 was deemed to be beneficial to humans and played an antagonistic role in the progression of metabolic diseases (21).

Recently, further research has proposed that omentin might be one of the salient adipokines intervening in the occurrence and progression of MAFLD, but the relationship between MAFLD and circulating omentin levels was inconsistent in different studies. Therefore, this study performed an integrative analysis of the circulating omentin levels in patients with MAFLD compared with healthy controls to reveal the association between omentin levels and MAFLD. This may lay the foundation for understanding the effects of omentin in MAFLD and provide new approaches for early diagnosis and identification of therapeutic targets of MAFLD.

## Methods

### Search strategy

This study was performed following the protocol published in PROSPERO (registration ID: CRD42022316369) and the Preferred Reporting Items for Systematic Reviews and Meta-Analyses (PRISMA) criteria (22). Two reviewers independently searched six databases: PubMed, Cochrane Library, EMBASE, CNKI, Wanfang, and CBM. Supplementary retrieval in the Clinical Trials Database (clinicaltrials.gov) and the Grey Literature Database (opengrey.eu) was also performed. The search was not limited by language and included publications up to April 8, 2022. Using the combination of subject terms and free-text words, the search query was formed as follows: (“Non-alcoholic Fatty Liver Disease” (MeSH) OR “NAFLD” OR “Nonalcoholic Steatohepatitis” OR “NASH” OR “Metabolic Associated Fatty Liver Disease” OR “MAFLD” OR “metabolic associated steatohepatitis” OR “MASH”) AND (“ITLN1 protein, human” (Supplementary Concept) OR “omentin” OR “intestinal lactoferrin receptor” OR “hIntL protein”). This query was slightly modified according to the different databases. A specific strategy is provided in **Supplementary Table 1**. If the full text or essential data of the included articles were not available, the corresponding authors were contacted *via* email for relevant information.

### Study selection

Two reviewers (QZ and SHC) independently assessed the eligibility of all studies, and any contradiction was resolved *via* discussion with a third reviewer (YNK). Studies were included if (1) patients were diagnosed with MAFLD, based on radiologically- proven hepatic steatosis and one of the three following conditions: overweight/obesity, diabetes mellitus, or metabolic dysregulation (e.g., HOMA-IR score ≥2.5); (2) the control group comprised healthy participants without MAFLD; (3) both groups included adults (age ≥18 years); and (4) outcomes included circulating serum or plasma omentin levels. Observational research cohort and case-control studies were included.

Studies were excluded if (1) patients had other causes of liver disease (e.g., alcoholic fatty liver disease or viral or autoimmune hepatitis) or severe disease; (2) omentin levels were not detected or were not derived from serum or plasma; (3) there were patient overlaps (i.e., more than one study conducted on the same patient cohort or duplicate publications); or (4) full-text or critical data of the articles was not obtained. Reviews, editorials, case reports, letters to the editor, hypotheses, studies on animals or cell lines, and abstracts from conferences were excluded.

### Data extraction and quality assessment

Two reviewers (QZ and SHC) independently extracted the relevant data and evaluated the quality of the included literature. If conflicts arose during the process, a third reviewer (YNK) adjudicated the disagreement between the two reviewers. The following data were extracted from the included studies: (1) basic characteristics of the studies (name of the first author, publication year, country where studies were conducted, and design), (2) criteria applied to diagnose and classify MAFLD, (3) methods used to detect circulating omentin levels, (4) characteristics of patient/control cohort (number, sex, age, body mass index [BMI], co-existing disorders including obesity and T2DM), (5) circulating omentin levels in serum or plasma, (6) biochemical measurements (aspartate aminotransferase [AST], alanine aminotransferase [ALT], total cholesterol [TC], total triglyceride [TG], low-density lipoprotein cholesterol [LDL-C], high-density lipoprotein cholesterol [HDL-C], C-reactive protein, interleukin-6, fasting serum insulin [FINS], fasting blood glucose [FBG]), and (7) calculation of homeostasis model assessment of insulin resistance [HOMA-IR].

The Newcastle–Ottawa Scale (NOS) is a useful tool for performing a semi-quantitative assessment of the quality of observational studies in a meta-analysis. The NOS contains eight items based on three dimensions: selection of cases, comparability of groups, and evaluation of exposures. Each item corresponds to one score, except that the item related to comparability corresponds to two scores. The quality of the studies is rated from 0 (very poor) to 9 (high) (23). The Grading of Recommendation, Assessment, Development, and Evaluation (GRADE) scale was used to assess the certainty of these studies (https://gdt.gradepro.org).

### Statistical analysis

Meta-analysis was performed using Stata/SE 15.1 (Stata Corporation, TX, USA). Omentin levels across each group were calculated as the *mean* ± *standard deviation* (*SD*). For omentin levels expressed as the *median* (*first quartile, third quartile*), the *mean* and *SD* were calculated using standardized formulas (24, 25). The standardized mean difference (*SMD*) was used to calculate the effect size in all studies. Heterogeneity among different studies was examined using the *I^2^* test, Cochran’s Q-test, and Galbraith’s test. A fixed-effects model was used for low heterogeneity (*P*≥0.1) and a random-effects model for high heterogeneity (*P*<0.1) (26). Subgroup and meta-regression analyses were conducted to explore the sources of heterogeneity. The subgroups were established based on the basic characteristics of the participants and physical indicators. Univariate meta-regression analysis was performed. The *SMD* of the omentin levels served as the dependent variable. The subgroup analysis variables and other biological indicators were added as explanatory covariates. Variables that met the significance level (*P ≤* 0.05) in the univariate analysis were entered into the multivariate meta-regression analysis. Additionally, a sensitivity analysis was conducted to assess the stability of the outcomes by sequentially excluding studies. Egger’s test and funnel plots were used to examine publication bias (27).

## Result

### Study selection

**Figure 1:**
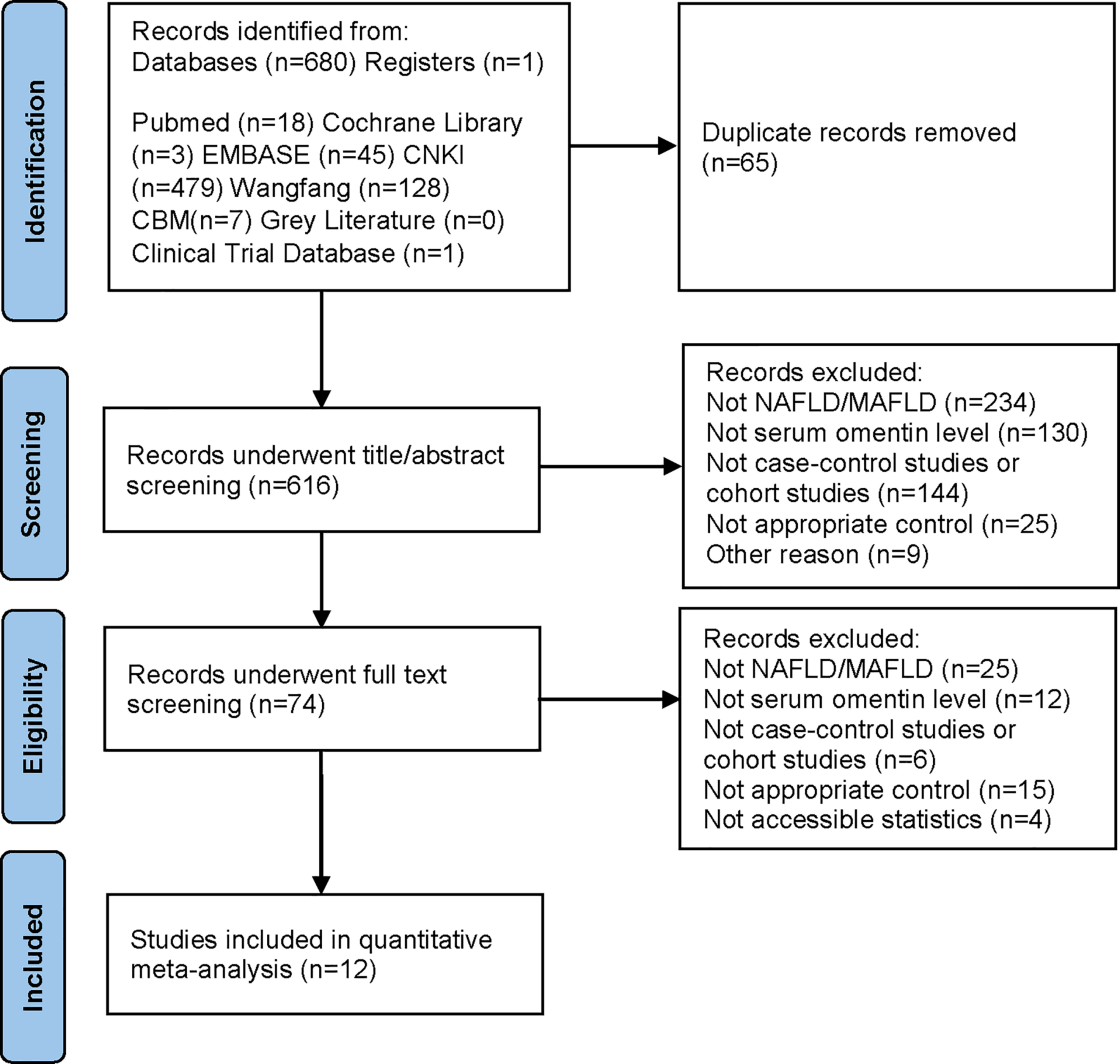
PRISMA diagram describing the process of literature search.

The entire literature search and selection process are presented in a PRISMA flow diagram (**Figure 1**). After retrieving articles from eight databases, 681 records were obtained. From the 681 records, 65 duplicates were removed, and the remaining studies were screened. Ultimately, 12 articles were included in the meta-analysis. The 12 articles were published between 2011 and 2021, reporting data on 1624 individuals (927 cases and 697 controls). Eight studies were conducted in China, one in Germany, one in Turkey, one in Poland, and one in Iran. The eight studies conducted in China were published in Chinese, and the remaining four were written in English. All articles were case-control studies that measured circulating omentin levels using enzyme-linked immunosorbent assays (ELISA). Seven of the twelve studies indicated that they evaluated omentin-1 levels, whereas the other five studies did not mention the specific subtype of omentin. The percentage of male subjects ranged from 31.71% to 100% in the patient groups and 38.76% to 100% in the control groups. The average ages of the patient and control groups were 51.51 and 51.26 years, respectively. BMI ranged from 26.1 to 41.0 kg/m^2^ in MAFLD cases and 21.35 to 27.4 kg/m^2^ in healthy controls. Supplementary specific information regarding the characteristics of the included studies can be found in **Table 1**.

**Table 1:** Baseline characteristics of studies included in the meta-analysis.

Author (year)	Country	MAFLD					Control					The type of omentin	Method of omentin measurement	Diagnostic methods
		No.	omentin (ng/ml)	sex (male%)	Age	BMI (kg/m2)	No.	omentin (ng/ml)	sex (male%)	Age	BMI (kg/m2)			
Bekaert et al. (28)	Germany	81	393.22 ± 131.03	67.90%	45 ± 10	41 ± 4.53	18	408.99 ± 124.13	100%	44 ± 12	24 ± 3.22	Omentin-1	ELISA	Liver Biopsy
Hang et al. (29)	China	200	42.38 ± 5.58	64.00%	45.41 ± 6.92	/	100	66.51 ± 8.24	62.00%	44.75 ± 6.32	/	Omentin-1	ELISA	Hepatic ultrasonography
Li et al. (30)	China	51	26.05 ± 7.26^a^	50.98%	59.54 ± 10.21	26.67 ± 1.45	49	22.9 ± 2.71^d^	48.98%	58.94 ± 11.35	21.35 ± 1.03	Omentin	ELISA	Hepatic ultrasonography or liver Biopsy
		50	17.85 ± 3.68^b^	52.00%	58.42 ± 11.86	26.01 ± 1.64					
Liu et al. (31)	China	30	35.69 ± 5.03	83.33%	65.4 ± 5.9	/	30	45.29 ± 6.25	80.00%	64.2 ± 5.4	/	Omentin	ELISA	Hepatic ultrasonography or liver Biopsy
Montazerifar et al. (32)	Iran	22	3.4301 ± 5.5791^a^	31.71%	39.8 ± 8.9	28.3 ± 4.2	33	2.9728 ± 4.456^d^	41.46%	36.7 ± 8.5	25.1 ± 3.6	Omentin-1	ELISA	Hepatic ultrasonography
		19	2.8574 ± 4.5415^c^	8			3.212 ± 5.2449^e^				
Shao et al. (33)	China	63	27.02 ± 2.82	44.44%	57.08 ± 10.5	26.73 ± 3.17	70	35.92 ± 2.8	48.57%	58.75 ± 9.71	23.47 ± 2.61	Omentin-1	ELISA	Hepatic ultrasonography
Waluga et al. (34)	Poland	25	266.6 ± 82.56	52.00%	31 ± 10	31.14 ± 6.07	25	114.5 ± 95.88	56.00%	42 ± 15	22.15 ± 0.83	Omentin-1	ELISA	Liver Biopsy
Wang and Wang (35)	China	138	3.29 ± 5.24	38.41%	43.83 ± 6.93	27.91 ± 4.64	129	4.56 ± 4.58	38.76%	42.72 ± 7.51	22.39 ± 2.78	Omentin-1	ELISA	Hepatic ultrasonography
Wang (36)	China	42	45.82 ± 21.46	42.86%	59.74 ± 9.48	26.14 ± 2.87	40	89.28 ± 18.04	57.50%	58.45 ± 10.48	22.53 ± 1.54	Omentin	ELISA	Hepatic ultrasonography or liver Biopsy
Yilmaz (37)	Turkey	99	460 ± 181	50.51%	48 ± 8	30.6 ± 4.9	75	376 ± 196	49.33%	48 ± 7	27.4 ± 4.3	Omentin	ELISA	Ultrasonography-guided liver biopsies
Yu et al. (38)	China	50	35.56 ± 5.03	100.00%	64.90 ± 5.59	27.06 ± 2.48	50	46.17 ± 6.53	100%	65.38 ± 6.34	25.62 ± 2.52	Omentin	ELISA	Hepatic ultrasonography or liver Biopsy
Zhang and Di (39)	China	57	36.86 ± 7.24	/	/	/	70	47.64 ± 7.24	/	/	/	Omentin-1	ELISA	Hepatic ultrasonography

Ranked by beginning letter of the first author. MAFLD, metabolic associated fatty liver disease; BMI, Body Mass Index; ELISA, enzyme‐linked immune‐sorbent assay; a. patients with MAFLD, b. patients with MAFLD and T2DM, c. patients with MAFLD and abdominal obesity; d. healthy controls; e. abdominal obesity.

### Quality assessment

The quality of the 12 included studies was evaluated using the NOS scale, with an average score of 5.58, which indicated that the overall risk of bias was moderate (**Table 2**). Nine studies were scored 6 or above, and the remaining studies were scored 4. Regarding the articles with a low score (<5), the studies conducted by Liu et al. and Wang (36) did not include community controls and lacked specific selection criteria for healthy controls (31, 36). The studies by *Waluga et al.* also did not include community controls and failed to meet the standard of representativeness (34). According to the GRADE scale (https://gdt.gradepro.org), the certainty of the present meta-analysis was very low (see **Supplementary Table 2**). Because all studies were observational, the certainty started at a low level. In addition, a low NOS score and significant heterogeneity led to a serious risk of bias and inconsistency, which combined with unmatched confounding factors between groups, accounted for a degradation of certainty.

**Table 2:** The Newcastle–Ottawa Scale (NOS) score of included articles.

No	author	year	Selection				Comparability	Exposure			Total	Average
			Adequate definition	Representativeness	Selection of Controls	Definition of Controls		Ascertainment of exposure	Same method	Non-response rate		
1	Bekaert, M. et al.	2016	1	1	1	1	0	1	1	0	6	5.583
2	Hang et al.	2019	1	1	1	1	0	1	1	0	6	
3	Li et al.	2012	1	1	1	1	0	1	1	0	6	
4	Liu et al.	2015	1	1	0	0	0	1	1	0	4	
5	Montazerifar, F. et al.	2017	1	1	1	1	0	1	1	0	6	
6	Shao et al.	2013	1	1	1	1	0	1	1	0	6	
7	Waluga, M. et al.	2019	1	0	0	1	0	1	1	0	4	
8	Wang et al.	2021	1	1	1	1	0	1	1	0	6	
9	Wang et al.	2013	1	1	0	0	0	1	1	0	4	
10	Yilmaz, Y.	2011	1	1	0	1	2	1	1	0	7	
11	Yu et al.	2013	1	1	1	0	1	1	1	0	6	
12	Zhang et al.	2016	1	1	1	1	0	1	1	0	6	

### Association between circulating omentin levels and MAFLD

**Figure 2:**
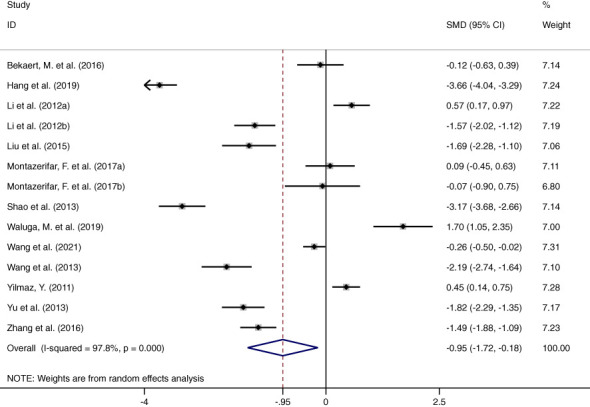
Forest plot presenting circulating omentin levels between MAFLD and healthy control group (Random-Effects Model, *SMD*). The omentin levels were significantly reduced in patients with MAFLD groups than in healthy controls.

**Figure 3:**
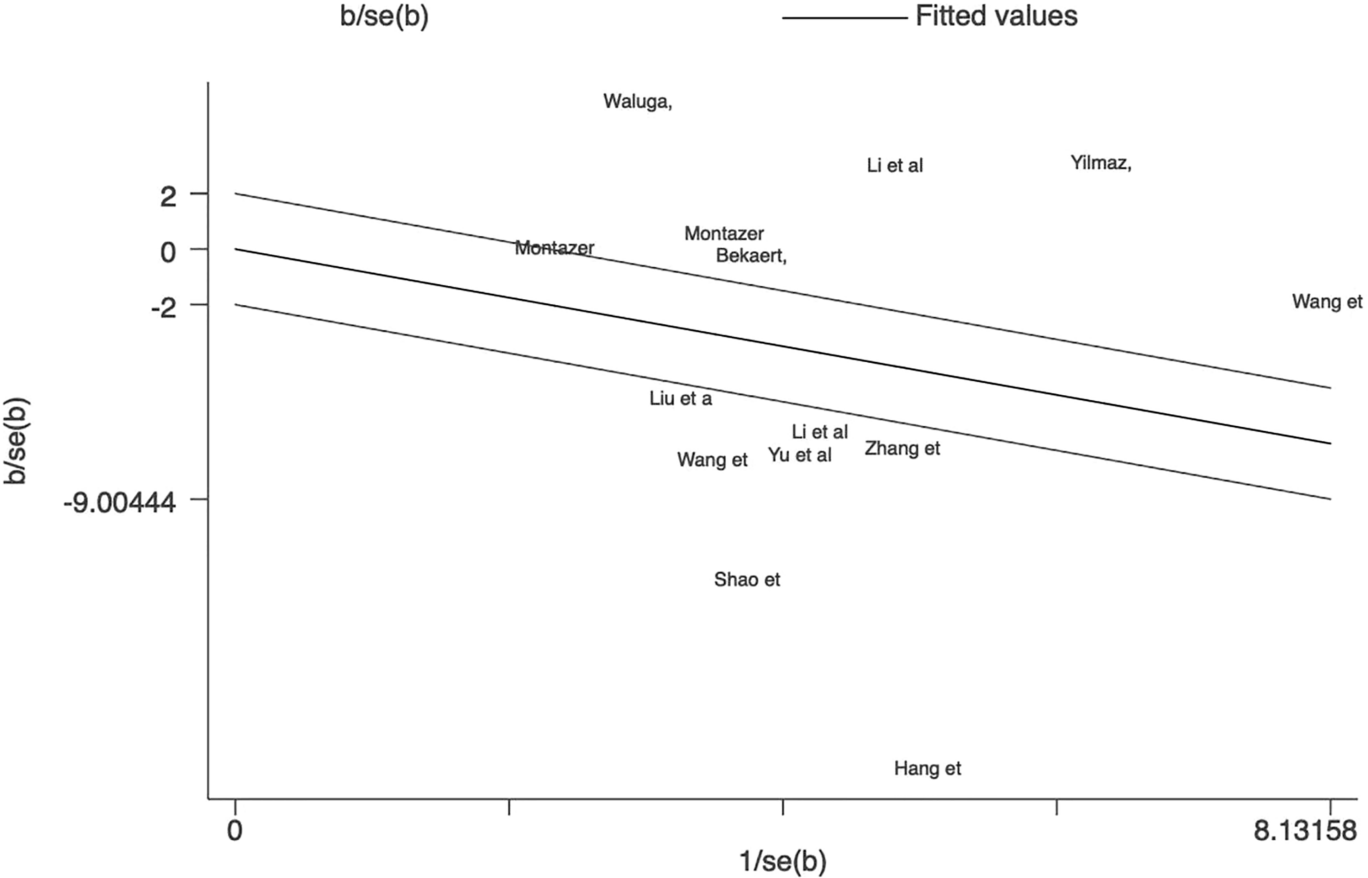
Galbraith test result of circulating omentin levels between MAFLD and healthy control group. Galbraith’s test showed that most of the studies were not within a reasonable range, which indicated significant heterogeneity between studies.

This meta-analysis examined 12 studies consisting of 14 comparisons of omentin levels between MAFLD and control groups (**Figure 2**) (28–39). Among these, the articles by Li et al. and Montazerifar et al. presented two statistical results (Li. et al.: MAFLD vs. controls and MAFLD+T2DM vs. controls; Montazerifar et al.: MAFLD vs. controls and MAFLD+abdominal obesity vs. abdominal obesity). The omentin levels were significantly lower in the MAFLD groups than in the control groups (*SMD*=-0.950 [-1.724, -0.177]). The random-effects model was applied because of its high heterogeneity (*P*<0.001, *I^2 =^* 97.8%). Obvious heterogeneity according to Galbraith’s test is also vividly exhibited in **Figure 3**, showing that most of the studies were not within a reasonable range. Therefore, subgroup analysis and meta-regression were necessary to further explore the sources of heterogeneity.

### Subgroup analysis

**Figure 4:**
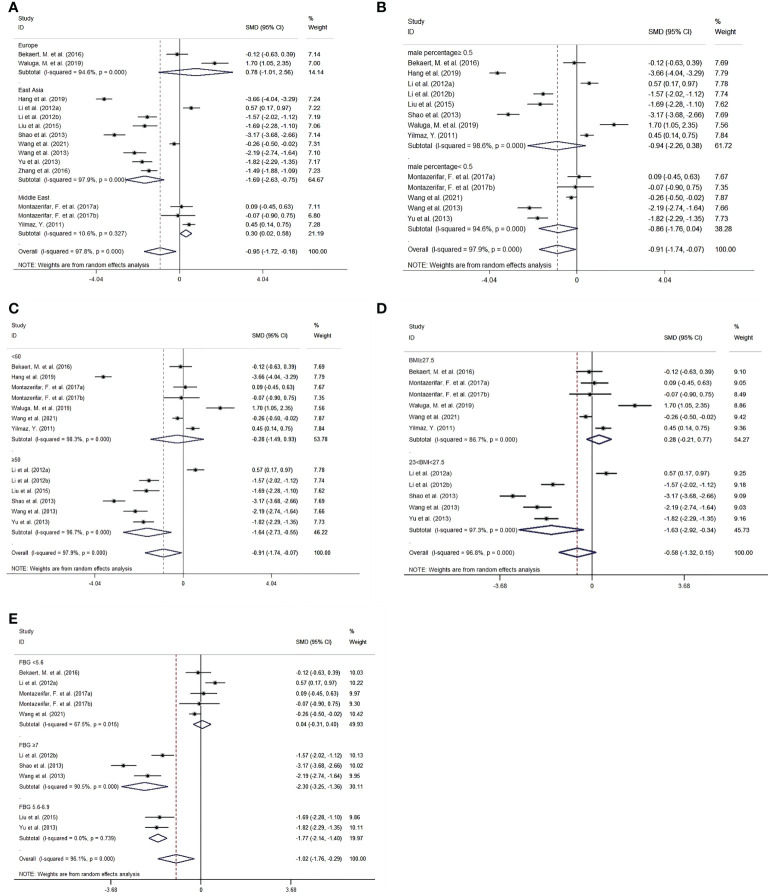
Forest plot for subgroup analysis of circulating omentin levels between MAFLD and healthy control group (Random-Effects Model, *SMD*). **(A)** subgroup analysis by region, **(B)** subgroup analysis by sex, **(C)** subgroup analysis by age, **(D)** subgroup analysis by BMI, **(E)** subgroup analysis by FBG.

Subgroup analysis was implemented by region, sex, age, BMI and FBG (**Figure 4**). As for subgroup analysis by region, in East Asia, omentin levels of patients with MAFLD were significantly lower than those of controls with an *SMD* of -1.69 [-2.63, -0.75]. In contrast, in the Middle East subgroup, patients with MAFLD had significantly higher omentin levels than healthy individuals with an *SMD* of 0.30 [0.02, 0.58]. There was no significant difference in omentin levels between patients with MAFLD and controls in the European subgroup, with an *SMD* of 0.78 [-1.01, 2.56]. It is worth noting that the heterogeneity in the East Asian and European groups were still high (*P*<0.001), and only that in the Middle East group decreased (*P*=0.327).

Regarding subgroup analysis by BMI. MAFLD patients with BMI between 23 and 27.5 kg/m^2^ had significantly lower omentin levels than controls (*SMD*=-1.63 [-2.92, -0.34]), whereas MAFLD patients with BMI ≥27.5 kg/m^2^ showed no significant difference compared with controls (*SMD*=0.28 [-0.21, 0.77]). Furthermore, patients with BMI between 23 and 27.5 kg/m^2^ had relatively lower omentin levels than those with BMI ≥27.5 kg/m^2^.

In addition, we performed subgroup analysis based on FBG. The cutoff values were based on Standards of Medical Care in Diabetes (40) (i.e., FBG ≥7 mmol/L for a diagnosis of diabetes and FBG of 5.6-6.9 mmol/L for impaired fasting glucose). In the subgroup with FBG <5.6 mmol/L, omentin levels were not significantly different between MAFLD patients and controls (*SMD*=0.04 [-0.31,0.40]). However, patients with FBG of 5.6-6.9 mmol/L or ≥7 mmol/L had significantly lower omentin levels than healthy controls (*SMD*=-1.77[-2.14, -1.40] and -2.30 [-3.25, -1.36], respectively). Intragroup heterogeneity was also reduced (FBG <5.6 mmol/L: *I^2 =^* 67.50%, *P*=0.015; FBG ≥7 mmol/L: *I^2 =^* 90.50%, *P*<0.001; 5.6 mmol/L≤ FBG<7 mmol/L: *I^2^* <0.001%, *P*=0.739). Other results of subgroup analysis by sex and age are listed in **Table 3**.

**Table 3:** subgroup analysis of circulating omentin levels between MAFLD and healthy control group.

Item	Number of comparisons^*^	*SMD [95% CI]*	Heterogeneity	
			*I^2^(%)*	*P* value
**Overall**	14	-0.95 [-1.72, -0.18]	97.80	<0.001
Subgroup analysis				
Region				
Europe	2	0.78 [-1.01, 2.56]	94.60	<0.001
East Asia	9	-1.69 [-2.63, -0.75]	97.90	<0.001
Middle East	3	0.30 [0.02, 0.58]	10.60	0.327
Sex				
Male percentage ≥0.5	8	-0.94 [-2.26, 0.38]	98.60	<0.001
Male percentage <0.5	5	-0.86 [-1.76, 0.04]	94.60	<0.001
Age				
Age <50	7	-0.28 [-1.49, 0.93]	98.30	<0.001
Age ≥50	6	-1.64 [-2.73, -0.55]	96.70	<0.001
BMI				
BMI ≥27.5	6	0.28 [-0.21, 0.77]	86.70	<0.001
23<BMI<27.5	5	-1.63 [-2.92, 0.34]	97.30	<0.001
FBG				
FBG<5.6	5	0.04 [-0.31, 0.40]	67.50	0.015
FBG≥7	3	-2.30 [-3.25, -1.36]	90.50	<0.001
5.6≤FBG<7	2	-1.77 [-2.14, -1.40]	<0.001	0.739

* Comparisons of omentin levels between MAFLD and control groups in included studies.

BMI, body Mass Index; CI, confidence interval; FBG, fast blood glucose; SMD, standardized mean difference.

### Meta-regression

Initially, 10 factors (region, sex, age, BMI, HOMA-IR, TC, TG, LDL-C, HDL-C, and FBG) were included in the univariate meta-regression. The results demonstrated that both region and FBG level exerted significant effects on the outcome. Region and FBG levels accounted for 39.22% and 78.47% of the between-study variance, respectively. Moreover, patients in Europe and the Middle East had higher omentin levels than those in East Asia. Patients with higher FBG levels had lower circulating omentin levels (Europe vs. East Asia: coefficient=2.467, *P*=0.024; Middle East vs. East Asia: coefficient=1.855, *P*=0.041; FBG: coefficient=-0.578, *P*=0.001). Region and FBG levels were then included in the multivariate meta-regression. The results showed that FBG level could better explain the heterogeneity, along with an inverse association with the *SMD* of circulating omentin levels (coefficient=-0.538, *P*=0.009) (**Table 4**).

**Table 4:** Meta-regression of the circulating omentin levels and MAFLD.

Covariates	Coefficient	Standard error	*t*	*P*	*95%CI*	
Univariate meta-regression analysis						
Area (reference= East Asia)						
Europe	2.467	0.941	2.62	0.024	0.396	4.537
Middle East	1.855	0.802	2.31	0.041	0.090	3.619
Gender	-2.095	2.315	-0.91	0.385	-7.190	3.000
Age	-0.078	0.037	-2.10	0.059	-0.160	0.004
BMI	0.129	0.102	1.27	0.235	-0.102	0.360
HOMA-IR	-0.183	0.294	0.62	0.552	-0.862	0.496
TC	0.515	0.770	0.67	0.520	-1.226	2.256
TG	-1.642	2.471	-0.66	0.525	-7.340	4.056
LDL-C	-0.109	1.145	-0.10	0.926	-2.749	2.531
HDL-C	7.069	3.751	1.88	0.096	-1.580	15.719
FBG	-0.578	0.109	-5.28	0.001	-0.830	-0.325
Multivariate meta-regression analysis						
Area (reference= East Asia)						
Europe	0.400	0.770	0.52	0.622	-1.485	2.285
Middle East	0.270	0.650	0.42	0.692	-1.320	1.859
FBG	-0.538	0.142	-3.79	0.009	-0.885	-0.190

BMI, body Mass Index; CI, confidence interval; FBG, fast blood glucose; HDL-C, high-density lipoprotein cholesterol; HOMA-IR, homeostasis model assessment of insulin resistance; LDL-C, low-density lipoprotein Cholesterol; TC, total cholesterol; TG, total triglyceride.

### Sensitivity analysis and publication bias

**Figure 5:**
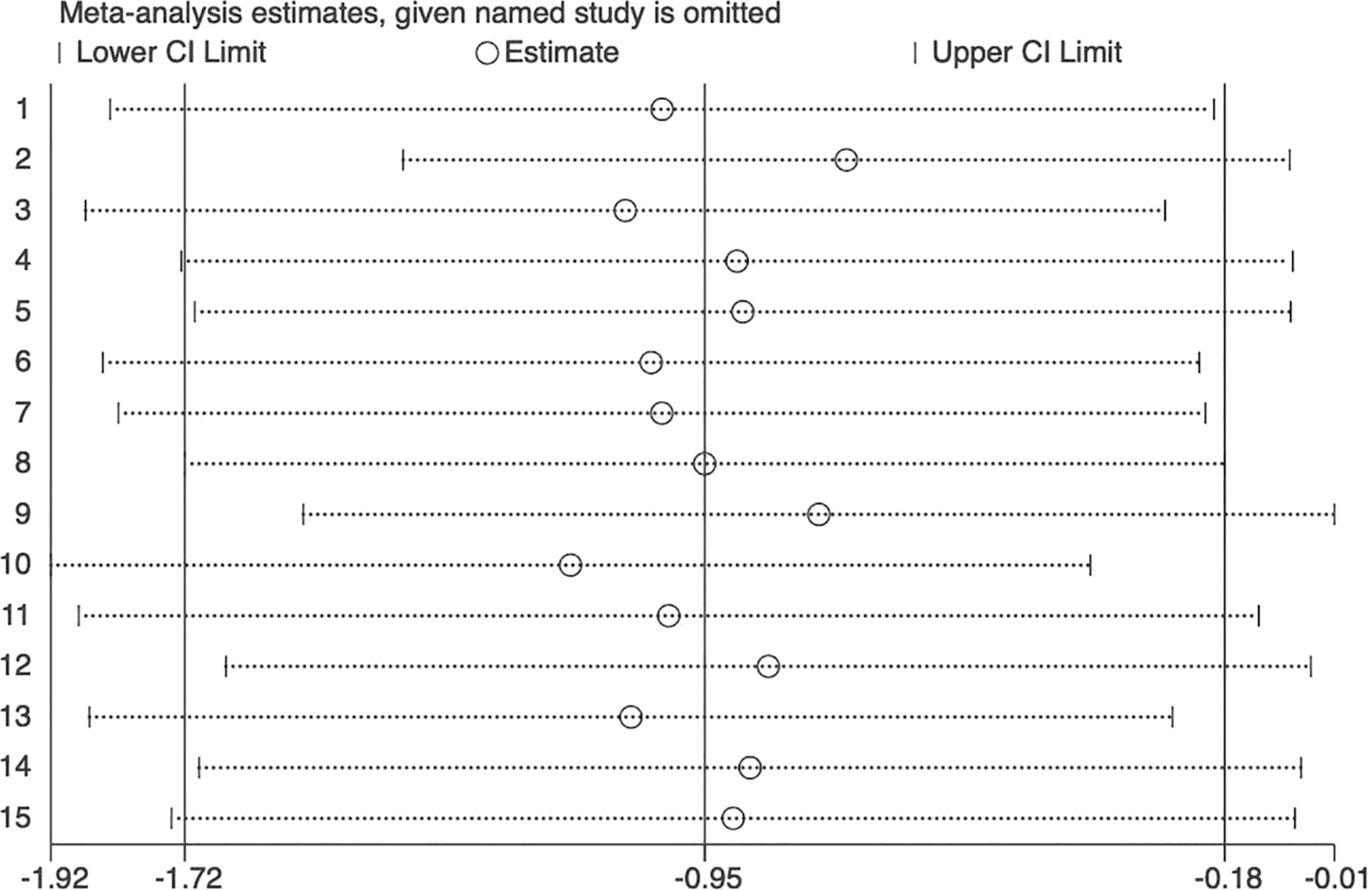
Sensitivity analysis plot of circulating omentin levels between MAFLD and healthy control group. Sensitivity analysis showed robustness of the outcomes and no single study overinfluenced the analysis.

**Figure 6:**
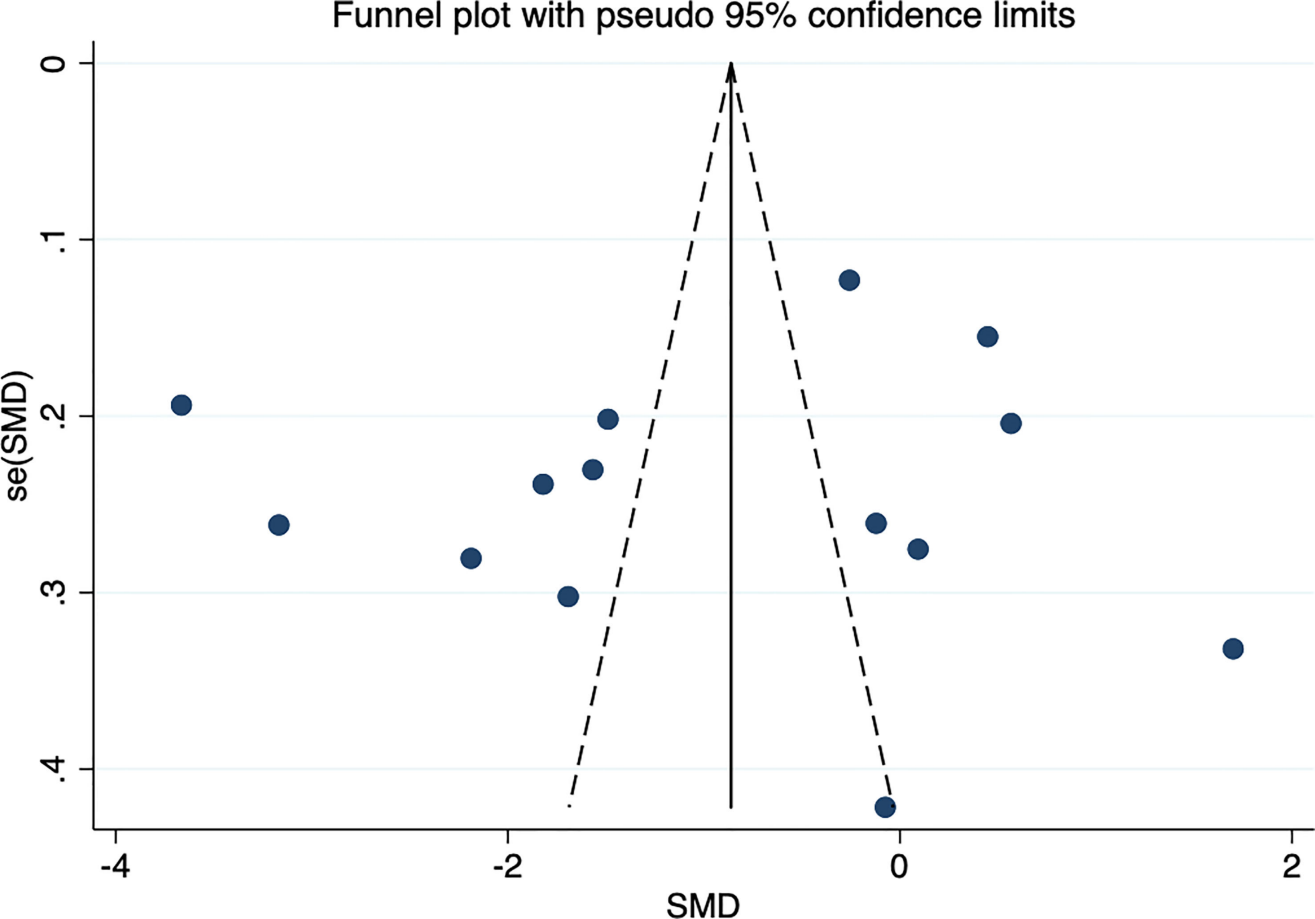
Funnel plot of circulating omentin levels between MAFLD and healthy control group. Funnel plot did not indicate a publication bias.

No study exerted a significant influence on the overall outcomes, which indicated benign stability (**Figure 5**). Egger’s test indicated a small possibility of publication bias (*P*=0.919). No obvious asymmetry was observed in the funnel plot, suggesting a low possibility of publication bias (**Figure 6**).

## Discussion

MAFLD, a multisystem disease, affects approximately one-quarter of the global population and brings about a considerable burden with wide-ranging social and economic implications (41, 42). The current situation urges us to seek a more effective approach for the management of MAFLD. At this time, metabolic insights into MAFLD provide new hope. Currently, the term MAFLD has replaced NAFLD to highlight the metabolic risk factors (43). Substantial research has indicated that MetS is bidirectionally related to MAFLD (44, 45). Not only does MetS increase the risks of MAFLD, but MAFLD also increases the symptoms and comorbidities of MetS (46). Moreover, this close association has been investigated in numerous studies for the advancement of research for diagnostic biomarkers and treatment agents. Experimental agents that target intermediary metabolism are likely to alleviate MAFLD symptoms and reduce cardiometabolic risk (47). Key biomarkers in the metabolic pathways of MAFLD have been identified using metabolomic and lipidomic methods for potential use in noninvasive diagnostic tests (48).

Omentin plays a crucial role in the maintenance of metabolism and insulin sensitivity (49). In the preclinical trials, omentin was found to significantly reduce fasting glycemia and ameliorate insulin resistance in rats with T2DM (50). Therefore, omentin administration could be a promising treatment for metabolic-related diseases, in addition to traditional lifestyle modification (51, 52). Moreover, clinical studies have shown that omentin levels in serum or visceral adipose tissue were significantly decreased in patients with NAFLD, MetS, T2DM, or obesity (11). Thus, the significant difference between the patients and healthy cohorts indicated that omentin could serve as a diagnostic biomarker for MAFLD. This kind of noninvasive measurement facilitates the clinician’s work, diminishes patient pain, and relieves the social burden. In general, considerable research has demonstrated that circulating omentin has excellent potential to be a noninvasive biomarker and treatment target for MAFLD (21). Therefore, it is of critical clinical value to explore the role of omentin in patients with MAFLD in this meta-analysis.

This meta-analysis included 12 studies, consisting of 1624 individuals (927 cases and 697 controls) from China, Germany, Turkey, Poland, and Iran. The pooled outcomes showed that circulating omentin levels were significantly lower in patients with MAFLD than in the healthy controls. The sensitivity analysis exhibited robustness of the results, and no significant publication bias was observed based on the Egger’s test and funnel plot. As heterogeneity was obvious between these studies, further subgroup analysis and meta-regression were necessary. In the subgroup analysis, studies in the Middle East and subgroups with FBG of 5.6-6.9 mmol/L showed no heterogeneity, whereas other subgroups divided by gender, age, and BMI showed high heterogeneity. Thus, the meta-regression analyzed more influential factors (including factors dividing the subgroups) to further explore the source of heterogeneity. Region and FBG both significantly affected the *SMD* of omentin levels in the univariate meta-regression, but the multivariate meta-regression revealed that FBG could better explain the high heterogeneity than region. In addition, the meta-regression failed to indicate a significant influence of other factors such as TC, TG, LDL-C, and HDL-C.

Regarding different regions, subgroup analysis showed that patients with MAFLD from East Asia had significantly lower circulating omentin levels, whereas patients with MAFLD in the Middle East showed significantly higher omentin levels than healthy controls. In contrast, there were no significant differences in omentin levels between patients with MAFLD and controls among European patients. Additionally, univariate meta-regression showed that patients from the Middle East and Europe exhibited higher omentin levels than those from East Asia. Given that high heterogeneity existed within the subgroups of different regions and insufficient studies were included in each region, future research is needed to verify the impact of region on circulating omentin levels.

Regarding FBG, in the subgroup analysis, omentin levels in patients with FBG <5.6 mmol/L showed no significant difference from controls, whereas patients with FBG of 5.6-6.9 or ≥7 mmol/L had significantly lower omentin levels. Meta-regression indicated an inverse relationship between FBG and the *SMD* of circulating omentin levels. To fully understand this relationship, we should bear in mind that FBG, insulin resistance, and MAFLD are closely associated. Insulin resistance, particularly hepatic insulin resistance, has been detected in subjects with impaired fasting glucose (53). Insulin resistance is one of the cardinal features of MAFLD and contributes to its development and progression (54). In addition to indirectly producing negative effects on MAFLD *via* insulin resistance, the increased FBG levels also directly elevate the risk of MAFLD (55, 56). In contrast, omentin, which decreases insulin resistance, is a protective factor against MAFLD (49). In summary, the occurrence and exacerbation of MAFLD are probably accompanied by increased FBG and decreased omentin levels. Moreover, Pan et al. observed that omentin was negatively correlated with FBG in a clinical trial, which is consistent with our outcomes (57).

BMI is another important factor that requires further investigation. In the subgroup analysis, because most of our included studies were conducted in Asia, we chose WHO-recommended Asian cutoffs of BMI (23.0-27.5 kg/m^2^ for overweight and ≥27.5 kg/m^2^ for obesity) as the basis for dividing subgroups (58). Previous studies found that the serum concentration and adipose tissue secretion of omentin were reduced in obese adults and adolescents (59). However, our subgroup analysis showed that omentin levels were lower in patients with a BMI between 23 and 27.5 kg/m^2^ than in those with a BMI ≥27.5 kg/m^2^. Patients with MAFLD and BMI between 23 and 27.5 kg/m^2^ had significantly lower omentin levels than controls, whereas omentin levels in patients with BMI ≥27.5 kg/m^2^ were not significantly different from those in controls. To explain it, we found that subgroup analysis by BMI was obviously influenced by regions. MAFLD cases with BMI between 23 and 27.5 kg/m^2^ were all from East Asia, and patients with BMI ≥27.5 kg/m^2^ were mostly from Europe and the Middle East. It has been reported that Asia had more non-obese MAFLD cases than other countries (60). For patients with non-obese MAFLD, steatohepatitis and fibrotic diseases were also observed even with normal BMI. Therefore, lower omentin levels were observed in patients with lower BMI, probably because omentin levels had a more direct relationship with MAFLD conditions than with BMI.

### Strengths and limitations

This meta-analysis investigated the association between circulating omentin levels and MAFLD. Subgroup analysis and meta-regression revealed that FBG levels might be a source of heterogeneity. The outcomes were reasonable and consistent, as previously discussed. Importantly, our study provides useful evidence for the clinical application of omentin as a biomarker to facilitate diagnosis or as a supplement to treatment.

This meta-analysis had certain limitations. First, the number of included studies and participants was small. Omentin is a novel adipokine that was identified in 2006, and relevant studies have been limited so far. Further studies are encouraged to investigate the association between omentin levels and patients with MAFLD, and an updated meta-analysis should be performed in the future. Second, the absolute value of omentin varied significantly from 2.85 to 460 ng/mL, although the units were uniform. Therefore, *SMD* and a random-effects model were chosen to pool the outcomes. Because all studies used ELISA to measure omentin levels, the distinctions between them might be derived from different experimental kits of different manufacturers. Third, the heterogeneity was high. We have conducted the subgroup analysis and meta-regression to explore the source of heterogeneity. Although we conducted subgroup analysis using as many variables as possible, they were still limited. The results demonstrated that FBG might be a possible source of heterogeneity, but several considered indicators (i.e., ALT, AST, FINS, and HbA1c) were not included in the meta-regression due to an insufficient number of studies that reported those indicators. Some of those indicators were essential to the association between omentin and MAFLD. Therefore, we hope that relevant articles in the future will pay more attention to multiple serum biomarkers. Furthermore, in order to fully identify the source of heterogeneity, different severities of MAFLD are another important factor to be considered. Since classification of the severity of MAFLD was rarely seen in the included studies, we could not perform a subgroup analysis based on disease severity.

## Conclusion

In conclusion, this meta-analysis demonstrated that circulating omentin levels were significantly lower in patients with MAFLD than in healthy controls. The FBG level, which had a significantly inverse relationship with circulating omentin levels, might be the source of heterogeneity. It should be noted that this conclusion might be more suitable for the Asian population, because Asian studies accounted for a large part of the meta-analysis. This meta-analysis might be conducive to clinical advances. Investigation of the association between circulating omentin and MAFLD could provide new insights into developing more convenient diagnostic biomarkers and effective treatment targets. Further research on omentin and MAFLD is necessary to confirm our conclusions.

## Data availability statement

The original contributions presented in the study are included in the article/**Supplementary Material**. Further inquiries can be directed to the corresponding authors.

## Author contributions

JH and SL: study design, quality assessment of the included studies and revision of the article. QZ and SC: data collection, performing the analysis and drafting the article. YK: quality assessment of the included studies and revision of the article. QL, CS, KW and YR: data collection and quality assessment of the included studies. All authors contributed to the article and approved the submitted version.

## Supplementary material

### Abbreviations

ALT, alanine aminotransferase; AST, aspartate aminotransferase; BMI, body mass index; CI, confidence interval; ELISA, enzyme-linked immunosorbent assays; FBG, fasting blood glucose; FINS, fasting serum insulin; GRADE, grading of recommendation, assessment, development, and evaluation; HDL-C, high-density lipoprotein cholesterol; HOMA-IR, homeostasis model assessment of insulin resistance; IL-6, interleukin-6; LDL-C, low-density lipoprotein Cholesterol; MAFLD, metabolic-associated fatty liver disease; MeSH, Medical Subject Heading; MetS, metabolic syndrome; NAFLD, non-alcoholic fatty liver disease; NOS, Newcastle–Ottawa scale; PRISMA, preferred reporting items for systematic reviews and meta-analyses; SD, standard deviation; SMD, standardized mean difference; T2DM, type 2 diabetes mellitus; TC, total cholesterol; TG, total triglyceride; VAT, visceral adipose tissue; WHO, World Health Organization.
