# Supplementary material for: Association of circulating omentin level and metabolic-associated fatty liver disease: a systematic review and meta-analysis
Source: Front Endocrinol (Lausanne). 2023 Apr 17;14:1073498. doi: 10.3389/fendo.2023.1073498 (PMC10150062; doi:10.3389/fendo.2023.1073498)
Supplement: Supplementary file 1 [file Table_1.docx]

| **Supplementary Table 1. Search Strategy** | | |
| --- | --- | --- |
| **Databases** | **Search terms** | **Number of records** |
| PubMed | #1 "Non-alcoholic Fatty Liver Disease"[Mesh] | 19,614 |
|  | #2 ((((((((((((((((((((((("Non-alcoholic Fatty Liver Disease") OR ("Non alcoholic Fatty Liver Disease")) OR ("NAFLD")) OR ("Nonalcoholic Fatty Liver Disease")) OR (Fatty Liver Nonalcoholic)) OR (Fatty Livers Nonalcoholic)) OR (Liver Nonalcoholic Fatty)) OR (Livers Nonalcoholic Fatty)) OR ("Nonalcoholic Fatty Liver")) OR ("Nonalcoholic Fatty Livers")) OR ("Nonalcoholic Steatohepatitis")) OR ("Nonalcoholic Steatohepatitides")) OR (Steatohepatitides Nonalcoholic)) OR (Steatohepatitis Nonalcoholic)) OR ("MAFLD")) OR ("Metabolic Associated Fatty Liver Disease")) OR ("Metabolic dysfunction associated fatty liver disease")) OR ("metabolic associated steatohepatitis")) OR ("MASH")) OR (Metabolically Associated Liver Steatosis)) OR ("Metabolic associated fatty liver") ) OR ("fatty liver")) OR ("NASH")) OR ("metabolic fatty liver")  #3 #1 OR #2 | 78,176 |
|  | #4 “ITLN1 protein, human" [Supplementary Concept] | 395 |
|  | #5 (((((((((((((("ITLN1 protein human") OR ("omentin")) OR ("omentin protein")) OR ("omentin 1 protein")) OR ("intelectin 1")) OR ("intelectin")) OR ("intestinal lactoferrin receptor")) OR ("hIntL protein")) OR (intelectin 1 protein human)) OR (omentin protein human)) OR (intelectin human)) OR (intestinal lactoferrin receptor human)) OR (hIntL protein human)) OR (intelectin 1 human)) OR (omentin 1 protein human)  #6 #4 OR #5 | 791 |
|  | #7 #3 and #6 | **18 results** |
| Cochrane Library | #1 MeSH descriptor: [Non-alcoholic Fatty Liver Disease] explode all trees | 1,359 |
|  | #2 ('Non alcoholic Fatty Liver Disease') OR ('Non-alcoholic Fatty Liver Disease') OR ('NAFLD') OR ('Nonalcoholic Fatty Liver Disease') OR ('Fatty Liver, Nonalcoholic') OR ('Fatty Liver, Nonalcoholic') (Word variations have been searched)  #3 ('Nonalcoholic Steatohepatitis') OR ('Nonalcoholic Steatohepatitides') OR (Steatohepatitides Nonalcoholic) OR (Steatohepatitis Nonalcoholic) OR ('MAFLD') (Word variations have been searched)  #4 ('Fatty Livers, Nonalcoholic') OR (Liver Nonalcoholic Fatty) OR (Livers Nonalcoholic Fatty) OR (‘Nonalcoholic Fatty Liver’) OR ('Nonalcoholic Fatty Livers') (Word variations have been searched)  #5 ('Metabolic Associated Fatty Liver Disease') OR (‘Metabolic dysfunction associated fatty liver disease’) OR (‘metabolic associated steatohepatitis’) OR (‘MASH’) OR (Metabolically Associated Liver Steatosis) (Word variations have been searched)  #6 ('Metabolic associated fatty liver') OR ('fatty liver') OR (‘NASH’) OR (‘metabolic fatty liver’) (Word variations have been searched)  #7 #1 or #2 or #3 or #4 or #5 or #6 | 7,882 |
|  | #8 (ITLN1 protein human) OR (‘omentin’) OR (‘omentin protein’) OR (‘omentin 1 protein’) OR (‘intelectin 1’) (Word variations have been searched)  #9 (‘intelectin’) OR (‘intestinal lactoferrin receptor’) OR (‘hIntL protein’) OR (intelectin 1 protein human) OR (omentin protein human) (Word variations have been searched)  #10 (omentin 1 protein human) (Word variations have been searched)  #11 (‘ITLN1 protein’) OR (intelectin human) OR (intestinal lactoferrin receptor human) OR (hIntL protein human) OR (intelectin 1 human) (Word variations have been searched)  #12 #8 or #9 or #10 or #11 | 93 |
|  | #13 #7 and #12 | **3 results** |
| EMBASE | #1 'nonalcoholic fatty liver'/exp | 54,549 |
|  | #2 'metabolic fatty liver'/exp | 431 |
|  | #3. 'fatty liver'  #4. 'non-alcoholic fatty liver disease'  #5. 'non alcoholic fatty liver disease'  #6. 'nonalcoholic fatty liver'  #7. 'nafld'  #8. 'nonalcoholic fatty liver disease'  #9. 'fatty liver, nonalcoholic'  #10. 'fatty livers, nonalcoholic'  #11. 'liver, nonalcoholic fatty'  #12. 'livers, nonalcoholic fatty'  #13. 'nonalcoholic fatty liver'  #14. 'nonalcoholic fatty livers'  #15. 'nonalcoholic steatohepatitis'  #16. 'nonalcoholic steatohepatitides'  #17. 'nash'  #18. 'steatohepatitides, nonalcoholic'  #19. 'steatohepatitis, nonalcoholic'  #20. 'metabolic fatty liver'  #21. 'mafld'  #22. 'metabolic associated fatty liver disease'  #23. 'metabolic dysfunction associated fatty liver disease'  #24. 'metabolic associated steatohepatitis'  #25. 'mash'  #26. 'metabolically associated liver steatosis'  #27. 'metabolic associated fatty liver'  #28. #1 OR #2 OR #3 OR #4 OR #5 OR #6 OR #7 OR #8 OR #9 OR #10 OR #11 OR #12 OR #13 OR #14 OR #15 OR #16 OR #17 OR #18 OR #19 OR #20 OR #21 OR #22 OR #23 OR #24 OR #25 OR #26 OR #27 | 120,125 |
|  | #29. 'omentin'/exp | 312 |
|  | #30. 'omentin 1'/exp | 286 |
|  | #31. 'omentin gene'/exp | 10 |
|  | #32. 'itln1 protein human'/exp | 46 |
|  | #33. 'intelectin'/exp | 37 |
|  | #34. 'intelectin 1'/exp | 65 |
|  | #35. 'omentin'  #36. 'omentin 1'  #37. 'omentin gene'  #38. 'omentin protein'  #39. 'omentin 1 protein'  #40. 'omentin protein human'  #41. 'omentin 1 protein human'  #42. 'itln1 protein human'  #43. 'itln1 protein'  #44. 'intelectin'  #45. 'intelectin 1'  #46. 'intestinal lactoferrin receptor'  #47. 'intelectin 1 protein human'  #48. 'intelectin human'  #49. 'intelectin 1 human'  #50. 'intestinal lactoferrin receptor human'  #51. 'hintl protein'  #52. 'hintl protein, human'  #53. #29 OR #30 OR #31 OR #32 OR #33 OR #34 OR #35 OR #36 OR #37 OR #38 OR #39 OR #40 OR #41 OR #42 OR #43 OR #44 OR #45 OR #46 OR #47 OR #48 OR #49 OR #50 OR #51 OR #52 | 1,299 |
|  | #54. #28 AND #53 | **45 results** |
| CNKI | FT='非酒精性脂肪性肝病' OR FT ='代谢相关脂肪性肝病' OR FT ='非酒精性单纯性脂肪肝' OR FT ='单纯性脂肪肝' OR FT ='非酒精性脂肪性肝炎' OR FT ='代谢相关脂肪性肝炎' OR FT ='非酒精性脂肪肝' OR FT ='非乙醇性脂肪肝' OR FT='非酒精性脂肪肝病' OR FT='非酒精性肝炎' OR FT='脂肪性肝炎' | 104,309 |
|  | FT='网膜素' OR FT ='网膜素-1' OR FT = '网膜素蛋白' OR FT ='内凝集素' OR FT ='内凝集蛋白' OR FT ='肠道乳铁蛋白受体' OR FT='omentin' OR FT='Intelectin' OR FT='ITLN1' OR FT='hIntL’ | 5,445 |
|  | ( FT='非酒精性脂肪性肝病' OR FT ='代谢相关脂肪性肝病' OR FT ='非酒精性单纯性脂肪肝' OR FT ='单纯性脂肪肝' OR FT ='非酒精性脂肪性肝炎' OR FT ='代谢相关脂肪性肝炎' OR FT ='非酒精性脂肪肝' OR FT ='非乙醇性脂肪肝' OR FT='非酒精性脂肪肝病' OR FT='非酒精性肝炎' OR FT='脂肪性肝炎') AND ( FT='网膜素' OR FT ='网膜素-1' OR FT = '网膜素蛋白' OR FT ='内凝集素' OR FT ='内凝集蛋白' OR FT ='肠道乳铁蛋白受体' OR FT='omentin' OR FT='Intelectin' OR FT='ITLN1' OR FT='hIntL') | **479 results** |
| Wanfang | 检索表达式（中英文扩展&主题词扩展）: 全部:(非酒精性脂肪性肝病 or 代谢相关脂肪性肝病 or 非酒精性单纯性脂肪肝 or 单纯性脂肪肝 or 非酒精性脂肪性肝炎 or 代谢相关脂肪性肝炎 or 非酒精性脂肪肝 or 非乙醇性脂肪肝 or 非酒精性脂肪肝病 or 非酒精性肝炎 or 脂肪性肝炎 or 代谢相关性脂肪肝病 or 代谢相关性脂肪肝) | 51,348 |
|  | 检索表达式（中英文扩展&主题词扩展）: 全部:(网膜素 or 网膜素-1 or 网膜素蛋白or 内凝集素 or 内凝集蛋白 or 肠道乳铁蛋白受体 or omentin or intelectin or ITLN1 or hIntL) | 59,110 |
|  | 检索表达式（中英文扩展&主题词扩展）： 全部:(非酒精性脂肪性肝病 or 代谢相关脂肪性肝病 or 非酒精性单纯性脂肪肝 or 单纯性脂肪肝 or 非酒精性脂肪性肝炎 or 代谢相关脂肪性肝炎 or 非酒精性脂肪肝 or 非乙醇性脂肪肝 or 非酒精性脂肪肝病 or 非酒精性肝炎 or 脂肪性肝炎 or 代谢相关性脂肪肝病 or 代谢相关性脂肪肝) and 全部:(网膜素 or 网膜素-1 or 网膜素蛋白or 内凝集素 or 内凝集蛋白 or 肠道乳铁蛋白受体 or omentin or intelectin or ITLN1 or hIntL) | **128 results** |
| CBM | (代谢相关性脂肪肝) OR (代谢相关性脂肪肝病) OR (非酒精性肝炎) OR (非酒精性脂肪肝病) OR (非乙醇性脂肪肝) OR (非酒精性脂肪肝) OR (代谢相关脂肪性肝炎) OR (非酒精性脂肪性肝炎) OR (单纯性脂肪肝) OR (非酒精性单纯性脂肪肝) | 16118 |
|  | (hIntL) OR (ITLN1) OR (intelectin) OR (omentin) OR (肠道乳铁蛋白受体) OR (内凝集蛋白) OR (内凝集素) OR (网膜素蛋白) OR (网膜素-1) OR (网膜素) | 526 |
|  | ((hIntL) OR (ITLN1) OR (intelectin) OR (omentin) OR (肠道乳铁蛋白受体) OR (内凝集蛋白) OR (内凝集素) OR (网膜素蛋白) OR (网膜素-1) OR (网膜素)) AND ((代谢相关性脂肪肝) OR (代谢相关性脂肪肝病) OR (非酒精性肝炎) OR (非酒精性脂肪肝病) OR (非乙醇性脂肪肝) OR (非酒精性脂肪肝) OR (代谢相关脂肪性肝炎) OR (非酒精性脂肪性肝炎) OR (单纯性脂肪肝) OR (非酒精性单纯性脂肪肝)) | **7 results** |
| Total |  | **680 results** |

**Supplementary Table 2. GRADE summary of findings**

| Outcome | Participants  (studies) | Risk of bias | Inconsistency | Indirectness | Imprecision | Other considerations | Overall certainty of evidence |
| --- | --- | --- | --- | --- | --- | --- | --- |
| Omentin | 12  Observational studies | Serious^1^ | Serious^2^ | Not serious | Not serious | Plausible confounding^3^ | VERY LOW |

^1^Serious risk of bias due to the NOS score (4-6)

^2^Serious inconsistency due to significant heterogeneity (I^2^>50%)

^3^All plausible residual confounding would reduce the demonstrated effect. Some of the studies do not match the basic information of two groups, like age, BMI and so on.
